# The Persian short form Aging Perceptions Questionnaire (APQ-P): A validation study

**DOI:** 10.1186/s12955-019-1259-x

**Published:** 2020-01-07

**Authors:** Mojgan Miremadi, Razieh Bandari, Majideh Heravi-Karimooi, Nahid Rejeh, Hamid Sharif Nia, Ali Montazeri

**Affiliations:** 1 Shahed University, Faculty of Nursing & Midwifery, Tehran, Iran; 2 Social Determinants of Health Research Center, Semnan University of Medical Sciences, Semnan, Iran; 3 Elderly Care Research Center, Faculty of Nursing& Midwifery, Shahed University, Tehran, Iran; 4 School of Nursing & Midwifery, Mazandaran University of Medical Sciences, Sari, Iran; 5 Population Health Research Group, Health Metrics Research Centre, Iranian Institute for Health Sciences Research, ACECR, Tehran, Iran; 6 Faculty of Humanity Sciences, University of Science & Culture, ACECR, Tehran, Iran

**Keywords:** Elderly, Aging perception questionnaire (APQ), Psychometric, Validity, Reliability

## Abstract

**Background:**

Population aging is an important phenomenon for many countries worldwide. Considering the growing trend of aging population in Iran, it is very important to consider beliefs and perceptions of old people about aging. The Aging Perception Questionnaire (APQ) is one of the most common instruments that used to measure aging comprehension. The present study was a methodological inquiry that aimed to examine validity and reliability of the Persian version of the Aging Perception Questionnaire (APQ).

**Methods:**

Forward-backward procedure was used for translation. Content validity and face validity were evaluated qualitatively. In order to evaluate construct validity a cross section study was conducted and both exploratory and confirmatory factor analyses were performed. In order to determine reliability, internal consistency (the Cronbach’s alpha) and stability (Intraclass Correlation Coefficient -ICC) were estimated.

**Results:**

A total of 500 elderly people attending the community centers in Tehran, Iran were entered in other study and completed the Persian version of the questionnaire. Most elderly were female (52.8%).The mean age of participants was 68.33 (SD ± 6.10) years. The results obtained from exploratory factor analysis showed a four-factors solution (consequences negative, emotional representation, control positive and consequences positive) that jointly explained 52.8% of the total variance observed. In addition the confirmatory factory analysis showed a good fit for the data. Finally, the Cronbach’s alpha coefficient of 0.83 ranging from 0.80 to 0.87 was obtained for the whole scale and the subscales. The ICC value of 0.96 ranging from 0.90 to 0.98 was found for the whole scale and the subscales.

**Conclusion:**

The results indicated that the Persian version of APQ is valid and can be used to measure aging perception in Iran.

## Background

Population aging is one of the most important social phenomena that are happening almost in all countries with different rates and paces [1]. The world’s elderly population is expected to rise by 25%, reaching 650 million in 2006 to 2 billion by 2050 [2].

Aging is a stage of the human life cycle, and based on different interpersonal relationships, people tend to form different perceptions of the biological, psychological, and social aspects of this phenomenon [3]. In order to understand people’s health status, feelings, personal identity, and behavioral outcomes in midlife and old age, it is essentially important to understand their experience and perceptions of aging [4].

Perception of ageing is referred to one’s perception of their own aging in the socio-cultural context they live in [5]. It is argued that perception of ageing is a criterion for aging satisfaction, and reflects level of adaptation to age-dependent changes [6]. However, perception of ageing varies in different stages of aging cycle (young old, middle old, and old old), and affects older adults’ behaviors and relationships with other people [5].

The factors influencing perception of aging at individual level include type of attitudes toward aging, mental age, gender, health status, financial status, marital status, religion, knowledge on aging, satisfaction with aging, and level of belief in internal locus of control. However it is believed that at social level the following factors could influence aging perception: modernism, culture, and social and family relationships [7]. As far as perception of aging relates to culture an extensive body of the literature exist. For instance in an scholarly paper McCann points out that while people in different cultures share some basic prototypes of aging perceptions, there are considerable inconsistencies about older people from one country to another [8]. A study on perceptions of aging across 26 cultures including countries from Asia and the West reported that variations in aging perceptions were associated with culture-level indicators of population aging, education levels, values, and national character stereotypes [9]. Thus, it seems that assessing aging perception in different countries is essential.

In terms of assessing perception of aging, a recommended instrument is the Aging Perceptions Questionnaire (APQ) that is a comprehensive and multidimensional questionnaire. It has been translated into different languages, and used in different countries, including the Netherlands [10], Brazil [11], China [12], France [13] and also among Turkish immigrants in the Netherlands [14]. Considering the importance of assessing perception of aging in the elderly population of Iran, and given the cultural differences between Iran and developed countries regarding aging, the present study aimed to translate the Aging Perceptions Questionnaire (APQ) from English into Persian and assess its psychometric properties in Iran. Like many other countries, population aging has an upward trend in Iran. The population of 60 years and above reached from 7.3% (more than 5 million) in 2006 [15–17] to 9.27% in 2016 (about 7.4 million) [18]. This is expected to reach 22% by 2046 [19].

## Methods

### The questionnaire

The APQ was developed by Barker et al. in Ireland [11]. This comprehensive and multidimensional instrument has two versions: a version containing 32 items and a brief version with 17 items [11, 20]. We used the former version that assesses 7 different dimensions, including timeline chronic (5 items), timeline cyclical (5 items), emotional representations (5 items), control positive (5 items), control negative (4 items), consequence positive (3 items), and consequence negative (5 items). The items are rated on a 5-point Likert scale, ranging from 1 (strongly disagree) to 5 (strongly agree) with the exception of the ‘control negative’ subscale, which are scored from 1 (strongly agrees) to 5 (strongly disagree). The higher scores indicate greater approval of a specific condition. We calculated the mean score for each subscales as recommended by the authors [11].

### Translation

Forward-backward translation procedure was used to translate the English version of the questionnaire into Persian. As such, two independent professionals translated the questionnaire from English into Persian. Then a consolidated Persian version of the two above-mentioned translations was provided with the best translation available. Subsequently two experts back translated the Persian version into English and it was compared with the original English version by the research team and the provisional version of the Persian version was provided.

### Content validity (qualitative content validity)

In order to examine content validity, 10 experts (two clinical psychologists, two psychiatrists, four assistant professors in nursing, and two assistant professors experienced in questionnaire design) were asked to qualitatively examine the questionnaire, and provide their opinions on the questionnaire in terms of grammar, vocabulary, necessity, importance, placement of the words, and scoring. The experts made no changes to the questionnaire.

### Face validity (qualitative face validity)

The APQ was administered to 10 older adults who met the inclusion criteria with maximum variance in order to assess the face validity of the questionnaire. Their views on appropriateness, difficulty, relevancy and ambiguity of the items were assessed. Almost all did not indicate any problems and thus the questionnaire was made ready for psychometric evaluation.

### Participants and the study setting

For the study purposes we thought at least 200 older adults (10 participants per item) are needed for exploratory factor analysis (EFA) and similarly 200 older adults are needed for confirmatory factor analysis (CFA) (21) [21]. In practice overall we recruited 500 older adults living in Tehran, Iran. Studies have shown that a sample from the general population in Tehran could at least be regarded as representative of the urban population of Iran [22, 23]. However, in Tehran there are 374 community centers located at different geographical areas (north, south, east, west and city center). The study samples were selected using a two stage stratified cluster random sampling method. First, all community centers were stratified according to the area (stratum). Then, of each stratum, proportional to population density between three to seven community centers were randomly selected (clusters). Data were collected from eligible participants at each cluster during two to 4 days to reach a predetermined sample size. All participants were asked to complete the study questionnaires in a calm setting. In the case of illiterate individuals the main investigator (MM) helped people to complete the questionnaires. In all instances completion of the questionnaires took about 15 min. The inclusion criteria were as follows: older adults aged 60 years and above, living in Tehran, ability to talk in Persian, not suffering from hearing loss or any mental or cognitive disorders (getting a score of 7 or higher on the Abbreviated Mental Test Score (AMTS) which was administered as part of this study. The AMTS is a relatively short cognitive screening tool [24]. It consists of 10 items, with one point given to each correctly answered question. The original AMTS puts the following questions to the patient: age (Item 1), time (to the nearest hour; Item 2), address for recall at end of the test (42 West Street; Item 3), year (Item 4), name of this place (Item 5), identification of two persons (doctor, nurse, etc.; Item 6), date of birth (Item 7), year of first world war (Item 8), name of the Queen (Item 9), and counting backwards from 20 to 1 (Item 10) [24]. In the Persian version of AMTS, the Item 8 had been changed to the year of Islamic Revolution, and the Item 9 to the name of the current leader of the country to make the test culturally and historically more appropriate. A score of 7 or less suggests probable cognitive impairment at the time of testing [25].

### Data analysis

The following analyses were performed in order to assess the psychometric properties of the questionnaire:

#### Construct validity

(i) In the first step of assessing construct validity, the exploratory factor analysis (EFA) was performed to extract latent factors. The Kaiser-Meyer-Olkin (KMO) test for sampling adequacy and the Bartlett’s test for sphericity were used. KMO values between 0.7 and 0.8 were considered as good, and values between 0.8 and 0.9 were considered as excellent [26]. Then, the latent factors were extracted using the maximum likelihood estimation, the varimax rotation, and scree plots. Presence of each item in the factor was determined according to communalities of above 0.5 in the EFA [27].

(ii) In the second step, the confirmatory factor analysis (CFA) was used to assess the most popular goodness of fit indices for the presented model according to the acceptable thresholds using the maximum likelihood estimation. Skewness ±3 and kurtosis ±7 were considered to indicate normal distribution [28]. Meyers at al. recommend the use of the following fit indices: The Chi-squared test (χ2)(CMIN), the Parsimonious Comparative Fit Index (PCFI), the Parsimonious Normed Fit Index (PNFI), the Root Mean Square Error of Approximation (RMSEA), the Goodness of fit index (GFI), the Adjusted Goodness of Fit Index (AGFI), and the Minimum Discrepancy Function by Degrees of Freedom (CMIN/DF) were examined [29]; we also used these indices.

(iii) Convergent and divergent validities were assessed using the average variance extracted (AVE), the maximum shared squared variance (MSV), and the average shared squared variance (ASV) Table 3. In order for the convergent validity to be stablished, the AVE should be above 0.5, and in order for the divergent validity to be stablished, the ASV and the MSV should be lower than the AVE [30].

#### Reliability

(i) In order to assess the internal consistency of the APQ, the Cronbach’s alpha coefficient was estimated first for the whole questionnaire and then for each extracted factor. An alpha value above 0.7 was considered to indicate good internal consistency [21]. Then, theconstruct reliability (CR) statistic for each the factors were assessed. CR values greater than 0.7 indicate good reliability and values between 0.6 and 0.7 can be accepted providing other indicators are good [21].

(ii) Stability was assessed using the intraclass correlation coefficient (ICC). When this index is above 0.75, there is a good level of stability [31]. Indeed a sub-sample of 30 elderly completed the questionnaire twice with a two-week an interval. The sub-sample was drawn randomly from the original sample consisting of 18 females and 12 males, with the mean age of 65.2 (SD = 4.8) years; 70% were married and most had primary or secondary education (*n* = 24). The remaining six participants (20%) had higher education. Although not the same, in general the characteristics of the sub-sample were very similar to the main participant pool. The SPSS version 18.0 and Amos 17.0 were used for statistical analysis.

## Results

In all 500 elderly took part in the study. Of these 372 (52.8%) were female, 68.0% (*n* = 340) were married, and 56.6% were retired. Most participants reported that they are living with family (36.4%) and indicated themselves as having intermediate economic status (41.6%). The characteristics of the participants are shown in Table 1.

**Table 1:** The characteristics of study participants (*n* = 500)

	Number (%)
Gender	
Man	236 (47.2)
Female	264 (52.2)
Age group (years)	
60–70	347 (69.4)
71–80	132 (26.4)
80<	21 (4.2)
Educational	
Illiterate	41 (8.2)
Primary	197 (39.4)
Secondary	149 (29.8)
Higher	113 (22.6)
Marital status	
Married	340 (68.0)
Single	13 (2.6)
Wideowed	138 (27.6)
Divorced	9 (1.8))
Employment status	
Housewife	175 (35.0)
Employed	35 (7.0)
Retired	290 (58.0)
Number of children	
0	24 (4.8)
1–3	226 (45.2)
4–6	223 (44.6)
> 7	27 (5.4)
Living condition	
Alone	103 (20.6)
With spouse	151 (30.2)
With children	59 (11.8)
With family	182 (36.4)
Others	5 (1.4)
Economic status	
Poor	135 (27.0)
Intermediate	208 (41.6)
Good	157 (31.4)
Housing	
Owner	398 (79.6)
Tenant	91 (18.20
Children’s home	6 (1.2)
Familiar home	5 (1.0)
Health status	
Very Poor/poor	26 (5.2)
Fair	189 (37.0)
Good/very good	285 (57.0)
History of the disease	
Yes	249 (49.8)
No	251 (50.2)

### Exploratory factor analysis

The KMO value in factor analysis model was found to be 0.86. In addition, the Bartlett’s test for Sphericity had a value of 4393.083, and was significant at 0.0001. The latent factors were extracted using the maximum likelihood estimation and the varimax rotation. In the model, four factors were extracted, based on eigenvalues above 1 and scree plots. As shown in Table 2, the factors jointly explained 52.8% of the variance observed. It is also worth to mention that the items 1–5 of timeline chronic, the items 27, 28, 30, and 32 of timeline cyclical, the items 14 and 15 of control positive, and the item 23 of control negative in the original version of the questionnaire were removed due to factor loadings below 0.3. The items of control negative, except the item 23, were put in the same category with the items of consequence negative. In addition, the item number 31 of timeline cyclical was replaced in the same category with the items of emotional representations (Table 2).

**Table 2:** Exploratory factor analysis of the APQ (*n* = 250)

Factor	Items	Factor load
Timeline Chronic		
	1. I am conscious of getting older all of the time	0.20
	2. I am always aware of my age	0.18
	3. I always classify myself as old	0.16
	4. I am always aware of the fact that I am getting older	0.06
	5. I feel my age in everything that I do	0.25
Timeline cyclical		
	27. I go through cycles in which my experience of ageing gets better and worse	0.15
	28. My awareness of getting older comes and goes in cycles	0.17
	30. I go through phases of feeling old	0.09
	32. I go through phases of viewing myself as being old	0.15
Control negative		
	23. I have no control over whether I lose vitality or zest for life as I age	0.24
**Consequences negative**		
	**16. As I age, I can not do some things**	**0.59**
	**17. Getting older makes me less independent**	**0.68**
	**18. Getting older makes everything a lot harder for me**	**0.63**
	**19. As I get older I can take part in fewer activities**	**0.76**
	**20. As I get older I do not cope as well with problems that arise**	**0.68**
	**21. Slowing down with age is not something I can control**	**0.64**
	**22. How mobile I am in later life is not up to me**	**0.60**
	**24. I have no control over the effects which getting older has on my social life**	**0.60**
**Emotional representations**		
	**9. I get depressed when I think about how ageing might affect the things that I can do**	**0.68**
	**13. I get depressed when I think about the effect that getting older might have on my social life**	**0.80**
	**25. I get depressed when I think about getting older**	**0.83**
	**26. I worry about the effects that getting older may have on my relationships with others**	**0.49**
	**29. I feel angry when I think about getting older**	**0.50**
	**31. My awareness of getting older changes a great deal from day to day**	**0.31**
**Control positive**		
	**10. The quality of my social life in later years depends on me**	**0.79**
	**11. The quality of my relationships with others in later life depends on me**	**0.87**
	**12. Whether I continue living life to the full depends on me**	**0.82**
	14. As I get older there is much I can do to maintain my independence	0.03
	15. Whether getting older has positive sides to it depends on m**e**	0.16
**Consequences positive**		
	**6. As I get older I get wiser**	**0.64**
	**7. As I get older I continue to grow as a person**	**0.94**
	**8. As I get older I appreciate things more**	**0.74**

Note. Items 21 to 24 are reverse coded. Items in bold are the items included in the Persian version of the APQ

### Confirmatory factor analysis

**Fig. 1:**
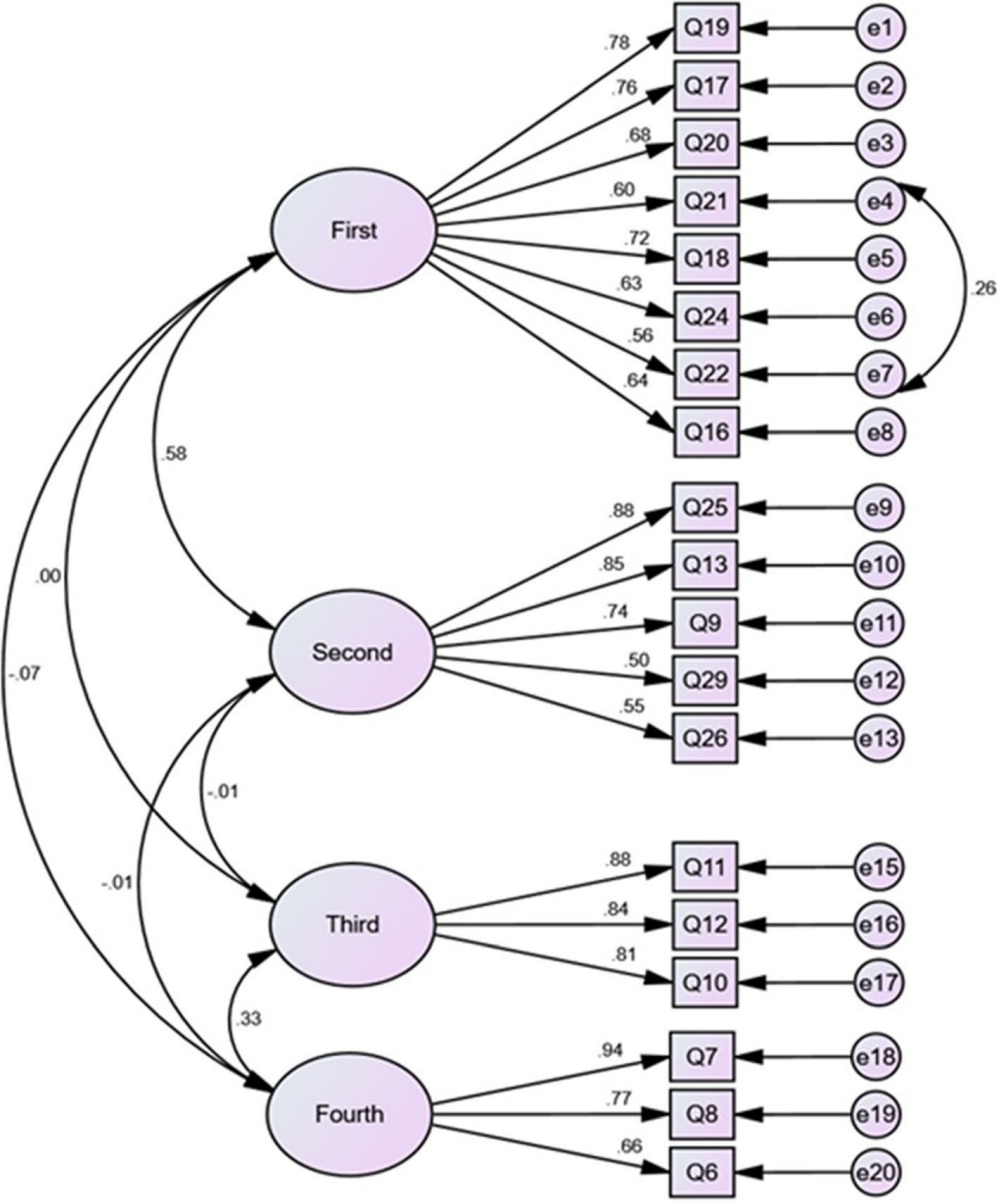
The results obtained from Confirmatory Factor Analysis (CAF) for the APQ-P. First (Consequences negative), Second (Emotional representations), Third (Control positive) and Fourth (Consequences positive)

The factor structure obtained with EFA was assessed and validated using maximum likelihood CFA with 250 participants. Based on the modification indices, one of measurement errors (between items 21 and 22) was allowed to freely co-vary (Fig. 1). Thus after reviewing model misfit, the single factor consisting of 19 items with good fit to the data was achieved. The fit indices were as follows: χ2 = 258.05, DF = 145, CMIN/DF = 1.78, RMSEA = 0.04, PCFI = 0.82, PNFI = 0.79 AGFI = 0.93 IFI = 0.97 CFI = 0.97. All values had acceptable thresholds and confirmed the hypothesized measurement model for the instrument (Table 3).

**Table 3:** The range of acceptable fit indexes of confirmatory factor analysis

Indexes	20 Items	32 Items
CMIN/DF	1.78	2.64
P	0.0001	0.0001
DF	145	443
GFI	0.97	0.80
AGFI	0.93	0.77
IFI	0.97	0.81
CFI	0.97	0.80
PCFI	0.82	0.72
PNFI	0.79	0.64
RMSEA	0.04	0.08

### Convergent and discriminant validity

According to the findings, factor AVE (0.54, 0.52, 0.70 and 0·638 respectively) was greater than MSV (0.33, 0.33, 0.10 and 0.10 respectively) and ASV (0.11, 0.11, 0.03 and 0.03 respectively). Therefore, the considered structure has appropriate convergent and divergent validity.

### Reliability

Finally, a Cronbach’s alpha of 0.83 ranging from 0.80 to 0.87 were found for the whole scale and the subscales, respectively. In addition, an ICC of 0.96 ranging from 0.90 to 0.98 were found for the whole scale and the subscales, respectively (Table 4) and CR (0.86, 0.83, 0.88 and 0·83 respectively) of the APQ in the four extracted factors in the present study was estimated to be desirable (*>* 0.7) (Table 5).

**Table 4:** The Cronbach's alpha and the Intraclass Correlation Coefficients (ICC) for the Persian version of the APQ

	Number of Items		Cronbach’s alpha		95% CI				ICC	
					Upper limit		*Lower limit*			
	20 Items	32 items	20 Items	32 items	20 Items	32 items	20 Items	32 items	20 Items	32 items
Consequences negative	8	5	0.87	0.81	0.99	0.83	0.97	0.82	0.98	0.82
Emotional representation	6	5	0.82	0.81	0.95	0.90	0.80	0.88	0.90	0.89
Control positive	3	5	0.87	0.33	0.96	0.54	0.82	0.38	0.92	0.46
Consequences positive	3	3	0.80	0.77	0.98	0.87	0.95	0.80	0.97	0.83
Timeline chronic	–	5	–	0.82	–	0.99	–	0.97	–	0.98
Timeline Cyclical	–	5	–	0.64	–	0.99	–	0.84	–	0.93
Control negative	–	4	–	0.84	–	0.99	–	0.96	–	0.98
Total	20	32	0.83	0.51	0.99	0.98	0.95	0.92	0.98	0.96

**Table 5:** Convergent and divergent values of the Aging Perception Questionnaire (APQ)

	*ASV*	*MSV*	AVE	CR
Consequences negative	0.11	0.33	0.54	0.86
Emotional representations	0.11	0.33	0.52	0.83
Control positive	0.03	0.10	0.70	0.88
Consequences positive	0.03	0.10	0.63	0.83

*AVS* Average Shared Variance, *MSV* Maximum Shared Variance, *AVE* Average variance extracted, CR: Construct Reliability

## Discussion

The present study was aimed to translate the Aging Perceptions Questionnaire (APQ) into Persian and assess its validity and reliability to be used in epidemiological and clinical studies. We used rigorous methods based on both psychometric and conceptual criteria. The final APQ-P was shorter than original one with improved fit indices relative to the long version. However, we kept the short version consistent with the original conceptual model. It covers the key dimensions of the ‘control positive’, ‘consequences negative’, ‘consequences positive’, and ‘emotional representations’. In addition reliability, in terms of internal consistency, was preserved in the Persian version. The convergent and discriminant validity also showed satisfactory results. However, one should note that we reduced 7 dimensions to 4 dimensions that, to some extent, is not unusual. To explain the issue further it is necessary to acknowledge that there are two APQ versions: one consisting of 7 dimensions with 32 items (APQ) and the second that is brief version containing 5 dimensions with 17 items (B-APQ). Now we introduced another version (APQ-P) that includes 4 dimensions with 20 items. Similarly studies on psychometric evaluation of the Turkish [14], and the Dutch versions [10] of the APQ reported removal of 11 items, although they kept the original 7 dimensions. However when performing exploratory factor analysis we observed that some items loaded onto different components compared to the original APQ. The item ‘slowing down with age is not something I can control’ and ‘How mobile I am in later life is not up to me’ ‘I have no control over the effects which getting older has on my social life’ related to ‘control negative’ was loaded onto ‘consequences negative’. The possible explanation is that most Persian old people live with family thus the old people in Iran might think that they increase family members’ burden and consequently they view slowing down as negative consequences of aging.

The results obtained from the EFA indicated that aging perception of the Iranian older adults had a multidimensional factor structure. Using the maximum likelihood and the varimax rotation, 4 factors were extracted that together explained 52.8% of the total variance. Similar results were reported by other investigators [12, 32]. Also, Slotman et al., by conducting an EFA, confirmed the multidimensional factor structure of the APQ [10, 14]. Similarly when performing CFA since all fit indices were in the acceptable range. The model had a good fit to the data and all the indices were satisfactory. The most commonly reported fit indices are: first, Chi-Square value, which is the traditional measure for evaluating the overall model fit and is affected by sample size; thus researchers have sought alternative indices to assess the model fit. Relative/normed Chi-square (χ2/df) minimizes the impact of sample size on the Model Chi-Square [30]. According to Kline; a model demonstrates the reasonable fit if the statistic adjusted by its degrees of freedom does not exceed 3.0 (χ2/df ≤ 3) [33, 34]. In this study, χ2/df was 1.78 The RMSEA is the second fit statistic reported in the AMOS program. An acceptable RMSEA is ≤0.1, and below 0.08 shows a good fit; and the CFI, GFI, AGFI, and the IFI, should be ≥0.90 [30].

The results of the present study showed that the items of the APQ enjoy appropriate convergent and divergent validity in its final model. In 2016 study, Hair states that convergent validity exists when the objects of the structure are close to each other and share alarge variance together. On the other hand, divergent validity is stated to exist whenthe items of the considered structure or the latent extracted factors are completelyseparate from each other [35]. In the clearer sense, the appropriate convergent validity would not be possible if the latent factors are not well explained by the extracted clauses and are not sufficiently correlated [36].

In the present study, overall Cronbach’s alpha of internal consistency reliability of the scale was 0.83, with 0.87 in consequences negative, 0.82 in emotional representation, 0.87 control positive and 0.80 in consequences positive dimensions. Sexton et al. found Cronbach’s alphas above 0.7 for all the subscales of the Brief Aging Perceptions Questionnaire (B-APQ), and proved its internal consistency [20]. Slotman and Cramm found an acceptable Cronbach’s alpha for the short version, indicating that the questionnaire had a good reliability [10]. Chen at al. assess the reliability of the Chinese version of the Questionnaire (C-APQ) and found acceptable alphas ranging from 0.665 to 0.869 for the subscales of the C-APQ [12]. Wang et al. found a Cronbach’s alpha of 0.87 for the APQ, indicating that the questionnaire had good consistency and reliability [32]. Using the APQ-S, the Slotman et al. study showed that all the subscales, except timeline cyclical, had good Cronbach’s alphas ranging from 0.75 to 0.88 [10]. Sadegh Moghaddam et al. found a Cronbach’s alpha of 0.75 for the total questionnaire (B-APQ), indicating that it had good reliability [5]. These results are all consistent with our results. In the present study also, CR was at a high level. One of the important attributes of CR estimation over Cronbach’s alpha is that it is not affected by the number of scale items and obtained structure and is dependent on the actual amount of factor load of each item on the latent variable. The CR value of the questionnaire was calculated in this study for the first time.

Stability was assessed using the test-retest analysis. There was a significant correlation that obtained from first and second assessments. This finding confirmed the repeatability of the questionnaire, and showed that the Persian version of the APQ had a good stability. Chen et al. found ICCs ranging from 0.82 to 1; this indicated that the APQ had a consistency [12]. A study assessed the reliability of the B-APQ, and found a correlation coefficient of 0.94, indicating that the questionnaire had good reliability [5]. Using the test-retest method with an interval of 2 months, Haghi et al. assess the reliability of the APQ. They reported significant coefficients for the two parts of the questionnaire and the whole questionnaire (*P* < 0.01) [37]. These results are also consistent with our findings.

### Strengths and limitations

Among the strengths of the present study were a large sample, random sampling, and performing confirmatory factor analysis. Some of the limitations include participants’ individual differences and different perceptions on the items of the questionnaire, superficial answers provided by some participants, cultural and class differences between the participants, and that only one researcher administered the questionnaire through interviews.

## Conclusion

The findings suggest that the Persian version of APQ has acceptable psychometric properties. Therefore, it can be used to measure aging perception in research and clinical settings.

## Data Availability

The datasets are available from the corresponding authors on request.

## Abbreviations

AGFI: Adjusted Goodness of Fit Index
APQ: Aging Perceptions Questionnaire
ASV: Average Shared Variance
AVE: Average Variance Extracted
CFA: Confirmatory Factor Analysis
CFI: Comparative Fit Index
EFA: Exploratory Factor Analysis
GFI: Goodness of Fit Index
ICC: Intraclass Correlation Coefficients
KMO: Kaiser–Meyer–Olkin
MSV: Maximum Variance
NFI: Normed Fit Index
NNFI: Non-Normed Fit Index
RMSEA: Root mean square error of approximation
SEM: Structural Equation Modeling
SRMR: Standardized Root Mean Square Residual
